# An Enhanced UV–Vis–NIR an d Flexible Photodetector Based on Electrospun ZnO Nanowire Array/PbS Quantum Dots Film Heterostructure

**DOI:** 10.1002/advs.201600316

**Published:** 2016-12-30

**Authors:** Zhi Zheng, Lin Gan, Jianbing Zhang, Fuwei Zhuge, Tianyou Zhai

**Affiliations:** ^1^ State Key Laboratory of Material Processing and Die & Mould TechnologySchool of Materials Science and EngineeringHuazhong University of Science and Technology (HUST)Wuhan430074P. R. China; ^2^ School of Optical and Electronic InformationHuazhong University of Science and Technology (HUST)Wuhan430074P. R. China

**Keywords:** flexible, heterostructures, photodetectors, ultraviolet*–*visible*–*near infrared, ZnO/PbS

## Abstract

ZnO nanostructure‐based photodetectors have a wide applications in many aspects, however, the response range of which are mainly restricted in the UV region dictated by its bandgap. Herein, UV–vis–NIR sensitive ZnO photodetectors consisting of ZnO nanowires (NW) array/PbS quantum dots (QDs) heterostructures are fabricated through modified electrospining method and an exchanging process. Besides wider response region compared to pure ZnO NWs based photodetectors, the heterostructures based photodetectors have faster response and recovery speed in UV range. Moreover, such photodetectors demonstrate good flexibility as well, which maintain almost constant performances under extreme (up to 180°) and repeat (up to 200 cycles) bending conditions in UV–vis–NIR range. Finally, this strategy is further verified on other kinds of 1D nanowires and 0D QDs, and similar enhancement on the performance of corresponding photodetecetors can be acquired, evidencing the universality of this strategy.

## 1 Introduction

Photodetectors with a broad spectral response from the ultraviolet (UV)–visible (vis) to the near infrared (NIR) has attracted great attention in a variety of applications, including image sensing, chemical/biological sensing, communication, day and nighttime surveillance.^1^, ^2^, ^3^, ^4^ 1D nanowires (NWs) have been widely applied in photodetecting because of their unique geometrical and electronic characteristics, which can provide large aspect‐ratio, direct pathways for charge transport.^5^, ^6^, ^7^ Assembling these NWs into highly ordered arrays as ideal building blocks can meet the miniaturization and functionalization requirements for future photoelectric devices.^8^ Although many strategies have been proposed,^9^, ^10^ assembling NWs into arrays effectively and inexpensively is still a challenge. Moreover, the response range of specific NWs is limited by their inherent bandgap, therefore, corresponding photodetectors are only sensitive to wavelengths in a relative narrow region.

Zinc oxide (ZnO) is emerging as a potential candidate in optoelectronic applications like light‐emitting diodes, laser diodes, and photodetectors,^11^ it has high photosensitivity for UV light due to its wide bandgap (3.4 eV).^12^ Some works have been done to broaden the response of ZnO from UV to wider range.^13^, ^14^, ^15^, ^16^, ^17^, ^18^, ^19^, ^20^ For example, Kouklin^15^ doped Cu impurities into ZnO NWs, which results in orders of magnitude enhancement of the spectral sensitivity over both UV and vis spectral ranges. Zhang et al.^16^ developed a photodetector based on a single ZnO/CdS core–shell wire, which exhibits an excellent response in a wide spectral range (372–548 nm). ZnO/Si branched NWs heterojunction photodiodes via growing ZnO nanowires on p‐type Si substrate show a 12.8 mA W^−1^ responsivity at around 900 nm.^17^ However, the doped structures have flaws in stability and reproducibility especially for p‐type doping,^18^ and the synthesis process for core–shell usually involves a complex adjustment and also the lattice matching should be considered for this structure.^19^, ^20^

Compounding narrow bandgap quantum dots (QDs) into ZnO is another strategy to expand the spectral response range.^21^, ^22^ Recently, PbS QDs have attracted attention for NIR detection applications because of their inherent narrow (1.3 eV)^23^ and tunable bandgap altered with size and composition.^24^, ^25^, ^26^ The broadening of sensitive spectral range of ZnO NWs by combination with PbS QDs has been demonstrated in solar cells.^27^ However, the study on the photodetection performance of such heterostructures is still rare. Moreover, to maintain the stability of QDs in solvents, very long and insulating ligands are used to cap the surface of QDs,^28^ which would hinder the charge transfer between NWs and QDs.^29^

Electrospinning is a facile and cheap method to fabricate various size of NWs on a large scale, which has been widely utilized for tissue regeneration, energy conversion and storage, and water treatment, etc.^30^, ^31^ Furthermore, through simple modification, electrospinning can achieve highly ordered nanowire arrays (NWA) on various substrates as well.^32^ Here, we fabricate UV–vis–NIR sensitive photodetector based on electrospun ZnO NWA/PbS QDs heterostructure which was realized by a one‐step ligand exchange in the 1,2‐ethanedithiol (EDT)/acetonitrile solution. Compared with pure ZnO NWA‐based photodetectors, the ZnO NWs/PbS QDs heterostructure photodetectors demonstrated superior performance, such as shorter rise (from 42 to 9 s) and decay time (from 22 to 2 s) and extended response range (from UV to vis–NIR region). Moreover, the heterostructure based photodetectors demonstrated a good flexibility and mechanical stability, maintaining a constant photoelectronic performance even after 200 cycles severely bending treatment.

## 2 Results and Discussion

### 2.1 The Morphology and Structure of the ZnO Nanowire Array/PbS QDs Film

**Figure 1:**
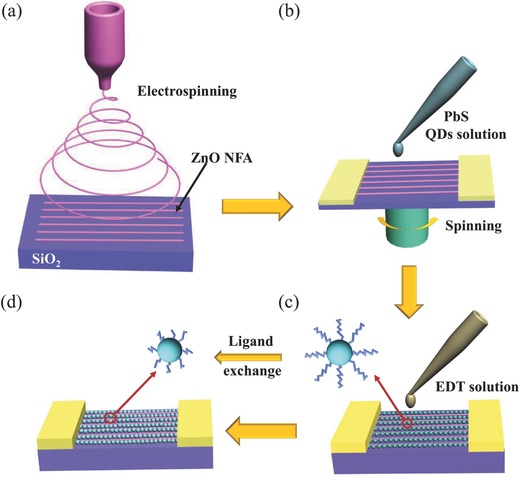
Schematic illustration of fabricating device for ZnO/PbS heterostructures. a) A schematic view for electrospinning ZnO NWA. b) A schematic spin‐coating PbS Qds on ZnO NWA. c,d) Process for exchanging OA ligand on the surface of PbS QDs in the EDT solution.

Fabrication of ZnO nanowire array/PbS QDs film (NWF) heterostructures are schematically depicted in experiment details as shown in **Figure** **1**. The fibers were stretched into a parallel array (Figure 1a) between the two separated electrodes on the collector and the mechanism has been elaborated in other works.^33^ ZnO NWA was obtained via a calcination process in air. This strategy was also suitable to fabricate other metal oxide NWAs, such as cadmium oxide (CdO) as demonstrated in Figure S1 (Supporting Information), suggesting enormous potential application in electronic integration. PbS QDs can form a thin film and attach to the surface and edge of ZnO NWs via a repeated spin*‐*coating process (Figure 1b). However, the spatial separation induced by the long oleic acid (OA) ligand that was used to cap QDs^34^ probably degrade the charge transfer between QDs and NWs. Therefore, it is necessary to exchange the long ligand with short one, such as EDT, tetrabutyl ammonium iodide (TBAI).^35^, ^36^, ^37^, ^38^ Herein, 1–2 mL EDT solution was dropped on the device to replace the long ligand of OA for about 2–3 min, see the schematic illustration in Figure 1c. As a result, dispersed QDs are then linked to a dense QDs film because of the ligand exchange.^39^ At the same time, heterojunction between QDs and ZnO NW was formed.^40^ Figure 1d shows the 3D scheme of the ZnO/PbS QDs hybrid phototransistor and the corresponding optical photograph is shown in Figure S2 (Supporting Information). The ZnO channel and the PbS QDs layers comprising the photodetector were clearly observed. Well‐aligned NWs are spanning the QDs film. Large particles with diameters outdistancing size of QDs are manifested in Figure S2 (Supporting Information), which results from the agglomeration of QDs produced during the repeated spin‐coating process. However, most QDs maintain their original morphology which has been confirmed by high‐resolution transmission electron microscopy (HRTEM) of PbS QDs thin film.

**Figure 2:**
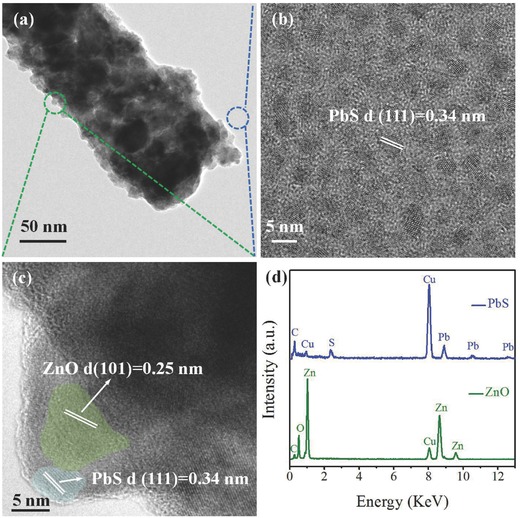
a) TEM for ZnO nanowires after compositing exchanged PbS QDs. b) HRTEM for PbS QD thin films after exchanging with EDT. c) HRTEM for ZnO nanowires after compositing exchanged PbS QDs. d) EDS spectra for treated ZnO nanowires and PbS QDs thin film, respectively.

To further investigate the as‐fabricated heterostructure, single exchanged ZnO NW was inspected by HRTEM as shown in **Figure** **2**a. The diameter of the NW is about 100 nm and is actually composed of agglomerated nanoparticles with diameters ranging from several nanometers to dozens of nanometers. Compared with pure ZnO NW, the rough surface after exchanging suggests the attachment of PbS QDs on the surface of ZnO NW. At the bottom of the NW, the HRTEM image of exchanged PbS QDs is depicted in Figure 2b. The QDs are dispersed uniformly on the carbon membrane. Size of PbS QDs is about 5 nm, and the 0.34 nm of marked lattice spacing is corresponded to the (111) planes. The distribution of composition for this heterogeneous structure is presented in the HRTEM image, it is clear that many grains with distinctively local parallel lattice fringes and interfaces at these grain boundaries can be observed in such ligand exchanged nanowires, displaying the formation of heterostructure and the simultaneous coexist of ZnO and PbS phases in an individual nanowire. The lattice‐fringe separation of 0.25 nm was identified as the (101) planes of hexagonal ZnO, (111) lattice of cubic PbS appeared in the ZnO NW indicated the attachment of PbS QDs after the process of exchange (Figure 2c). The element information was given in the energy dispersive X‐ray analysis spectrum (EDS) spectrum (Figure 2d). The blue spectrum is corresponding to the constituent of the film, which means that PbS QDs are dispersed in the area of blue circle. When the spot (green circle) is applied on the nanowires, the analysis indicates the presence of zinc, oxygen, copper, and carbon elements. High content of zinc and oxygen indicates the primary composition of ZnO nanowire after exchanging, the slight carbon signal might come from the tiny residue of amorphous carbon in the sintered NW and supporting carbon film on the transmission electron microscopy (TEM) grid. The copper signal comes from the TEM grid. The results of TEM indicate that PbS QDs were incorporated into the surface of ZnO NW, and formed a ZnO/PbS heterojunctions.

### 2.2 Optoelectronic Properties of ZnO/PbS NWF

**Figure 3:**
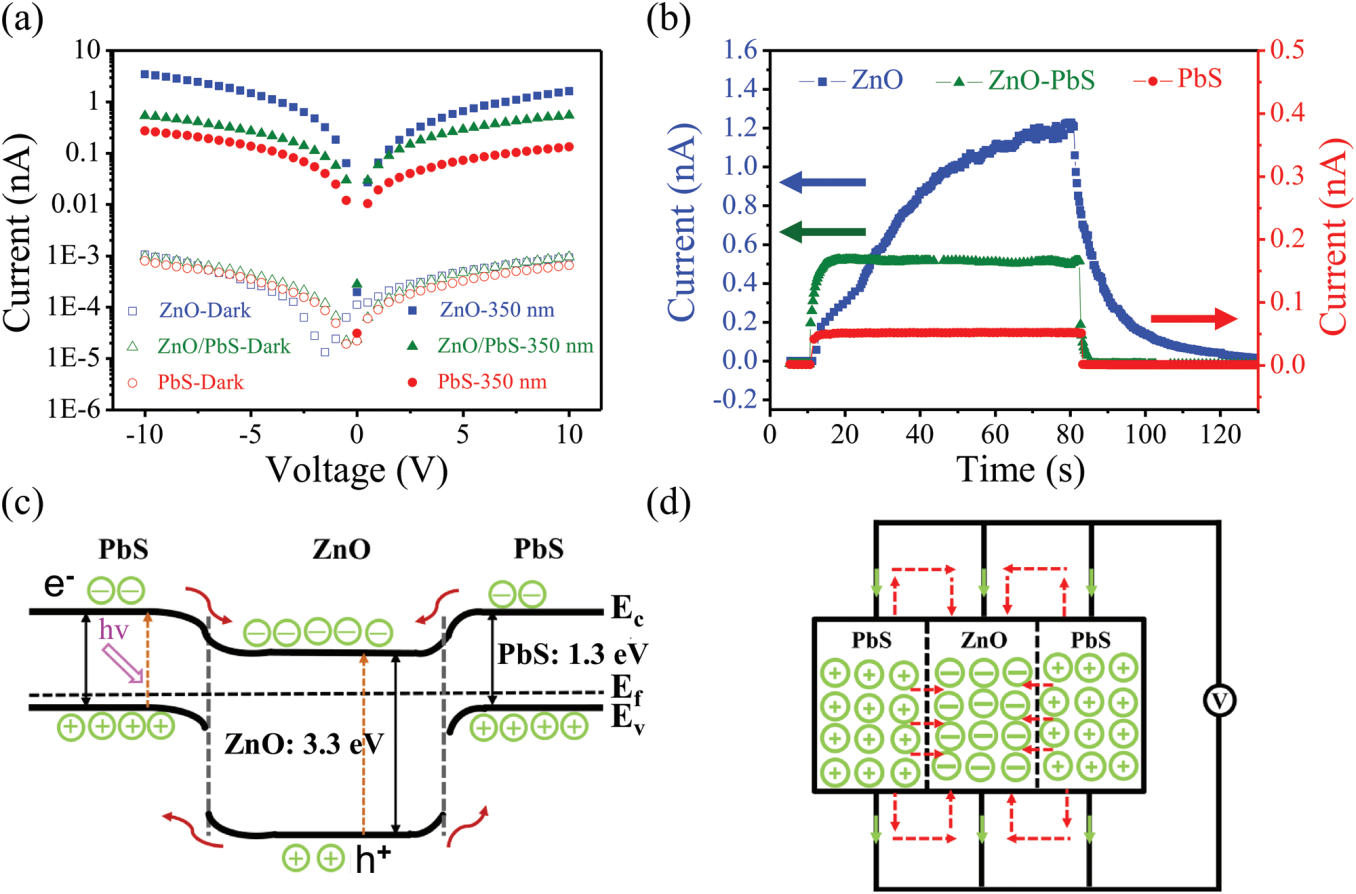
a) Current–voltage (*I*–*V*) curve for ZnO nanowires, ZnO/PbS heterostructure, and PbS thin film, in the dark and 350 nm illumination, respectively. b) Current–time (*I*–*T*) curve of the three types of devices during on–off switching tests at illumination of 350 nm, the bias is 10 V. c) Energy band diagram for the p–PbS/n–ZnO heterostructure. d) A schematic diagram of carrier transport condition for the ZnO NWA and PbS QDs film.

To further investigate the variation of photoelectric performance after decorating PbS QDs, two‐electrode devices configuration were fabricated based on ZnO NWA, ZnO/PbS heterostructures and PbS QDs thin film, respectively. Optical photograph of the device of heterostructure is shown in Figure S3a (Supporting Information). As ZnO has a high responsivity in the UV wavelength according to its bandgap (3.4 eV), a 350 nm light was chosen to excite the ZnO/PbS NWF to investigate the variation of photoelectric performance after decorating PbS QDs. The current*–*voltage (*I–V*) characteristics under dark and 350 nm illumination of the pristine ZnO NW before and after PbS QDs decoration and PbS thin film are shown in **Figure** **3**a and Figure S4 (Supporting Information). Both the pristine ZnO NW and PbS QDs‐decorated ZnO NW exhibit an increase in current upon exposure to UV light. Further, it should be noticed that the photocurrent of PbS QDs thin film is about ten times enhanced under the 350 nm light after exchange with EDT, which means that strategy for this exchange is necessary for property improvement (Figure 3b; Figure S5, Supporting Information). Compared to pristine ZnO NWs, PbS QDs‐decorated ZnO NWs exhibit a decrease of photocurrent. To calculate the optoelectrical properties^41^ of the ZnO/PbS photodetector, the responsivity (*R*) was calculated to be 0.051 A W^−1^ using the formula

(1) $$R=\frac{\Delta I}{PS}$$

(where Δ*I* = *I*_on_ – *I*_off,_ *V*_bias_ = 10 V, λ = 350 nm, *P* = 7.02 mW cm^−2^, refers to the incident light intensity and *S* = 1.5 × 10^−7^ cm^2^, is the effective area under illumination). Furthermore, detectivity (D*), an index to evaluate the photoresponse, can be expressed as follows

(2) $$D^{*}=\frac{R}{{\left(2qI_{\mathrm{dark}}/S\right)}^{1/2}}$$

where *I*_dark_ is the dark current, *S* is the effective area of the detectors, and *q* is the absolute value of electron charge (1.6 × 10^−19^ C). Then, the D* for this photodetector is calculated to be 3.4 × 10^8^ Jones. This value is comparable to the ZnO based thin film photodetectors,^42^ and the relatively high detectivity mainly comes from the low dark current, determined by the granular structures as shown in the HRTEM image. Grain boundaries existed in the NWs serve as energy barriers for carrier transport in dark condition, as a result, the dark current of the granular photodetector can be greatly suppressed.^43^ Photoelectric properties for ZnO NW and PbS QDs thin film are also calculated, the *R* and D* for ZnO NWA are 0.15 A W^−1^ and 9.9 × 10^8^ Jones; while for PbS QDs thin film, the *R* is 3.6 × 10^−6^ A W^−1^, the D* is 2.4 × 10^4^ Jones, respectively. The photoelectric performance for heterojunction is slightly inferior than the original ZnO NWA, which should be owning to the structure of device and will be explained below. In addition, comparison of response speed is demonstrated in Figure 3b. The rise time (*t*_r_) taken for the current to increase from 10% to 90% of the peak value or vice versa is defined as the decay time (*t*_d_), respectively.^44^ After compositing with PbS QDs, the rise time is significantly decreased to 9 s from original 42 s and the decay time decreased from 22 to 2 s. The origin of accelerating response speed by incorporating PbS QDs at the surface of ZnO is stated below. Oxygen adsorption on NW surface plays a central role in regulating the high photogain of a single ZnO NW photodetector.^45^ However, the process for adsorption and deadsorptoion of oxygen is relatively slow, in addition, surface of the NW has a complex defect composition, such as oxygen vacancy, which will hinder the transport of electron, lead to a slower process for surface photoconduction.^46^ By joining p‐type PbS QDs at the surface of n‐type ZnO NWs, nanoscale p−n heterojunctions are formed between PbS QDs and ZnO NWs, an electric field forms between the positive ion cores in the n‐type ZnO NWs and negative ion cores in p‐type PbS QDs, which results in the upward band bending from ZnO to PbS. A type‐II heterostructure with a staggered alignment at the heterojunction is formed for our ZnO/PbS NWF as shown in Figure 3c.^47^ This heterojunction would increase the width and the surface band bending region and as well as barrier height. Then, the thickness of the depletion layer will increase, the conductivity was mainly determined by the inner carriers, which is a bulk dominated process.^48^ In the ZnO/PbS heterostructure photodetector, electron–hole pairs are generated upon irradiation. The electrons in the depletion region migrate to the inner part of ZnO nanowire, while the holes drift to the surface of ZnO nanowire or even to the PbS layer, which discharge the negatively charged oxygen molecules adsorbed on NWA surface. As a result, unpaired electrons which contribute to the photocurrent are collected at the electrodes. However, the conducting electrons can also be captured or scattered by surface defects including native oxygen vacancy and dislocations result from lattice mismatch between ZnO and PbS, remaining photogenerated carriers in the NW core would have a higher mobility than the surface electrons. According to the relaxation dynamics in a ZnO NW photodetector, the carrier transport process includes bulk and surface transport mechanisms.^48^ After decorating PbS QDs, the interaction of photocarriers with oxygen molecules was minimized due to surface passivation, the proportion of bulk conduct was increased. Therefore, the response speed after decorating is much faster than the original ZnO NW.^46^ Generally, the spatial electron−hole separation effect^49^ under 350 nm light illumination is pronounced, then the recombination rate of electrons and holes is further reduced for a PbS QD‐decorated ZnO NW photodetector, leading to an increase in photocurrent. The reason for the decrease of photocurrent may be explained as follows: In this parallel structure of NWF device, ZnO nanowire and PbS QDs film are connected to each other at the electrodes, and contact between the NWs and QDs, that form a shorted p–n junction,^50^ the schematic diagram is described in Figure 3d. Photogenerated electrons and holes will recombine at the interface of the two nanomaterials and electrodes at the same time, resulting the decrease of photocurrent. Another reason may be that PbS QDs on the substrate impede the absorption of ZnO NW for UV light.

**Figure 4:**
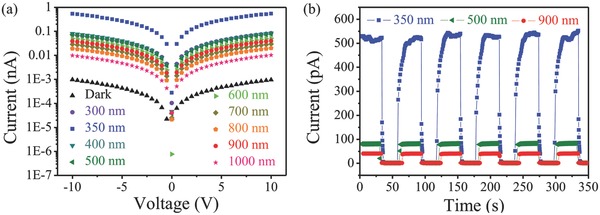
a) *I*–*V* curves of the device illuminated with light of various wavelengths and in the dark. b) *I*–*T* curves of the three types of devices during on–off switching tests under 350, 500, and 900 nm illumination.

ZnO NW is sensitive to UV light, and PbS QDs have a light sensitivity from UV/vis to NIR due to its bandgap of 1.3 eV (corresponding to 930 nm). The spectral response for this heterostructure should be determined by these two structures collectively.^51^ We further studied heterogeneous photoresponse with incident light of various wavelengths from UV to NIR, the photocurrent curves under dark conditions, 300, 350, 400, 500, 600, 700, 800, 900, and 1000 nm light are depicted in **Figure** **4**a, which have power densities to be 6.54, 7.02, 6.49, 4.12, 2.93, 2.25, 1.88, 2.55, and 3.26 mW cm^−2^, respectively. As seen from Figure 4a, the difference of power densities is much less than the difference of corresponding photocurrents, which evidence that the difference of photocurrent should be attributed to the selectivity of device on wavelengths rather than on the variation of power densities. In addition, these *I*–*V* curves under illumination exhibit linear behaviors as demonstrated in Figure S6 (Supporting Information), indicating a good ohmic contact between the NWF and the electrodes. The dark current was as low as 1 pA at the bias of 10 V, which was determined by the polycrystalline structure of the ZnO NWs. A large photocurrent can clearly be observed in these nine wavelengths. Even for the photocurrent under 1000 nm irradiation, it was still ten times higher than that under dark conditions. In other words, the photoresponses of this heterostructure span the full range from UV–vis to NIR, indicating the application potential as UV*–*vis*–*NIR detector. The photoresponse stabilities of devices were investigated under three typical given lights^52^ 350, 500, and 900 nm crossing over UV−vis−NIR range, as shown in Figure 4b. Light intensities of 350, 500, and 900 nm lasers were set to be 7.02, 4.13, and 2.55 mW cm^−2^, respectively. According to the Equations (1) and (2), the responsivities of 350, 500, and 900 nm are calculated as 0.051, 0.0072, and 0.011 A W^−1^, and the detectivities of these three wavelengths are calculated as 3.4 × 10^8^, 4.9 × 10^7^, and 4.2 × 10^7^ Jones, respectively. Device showed the highest response at 350 nm light which corresponds to the bandgap of ZnO and the second highest response at 900 nm light that is closed to the bandgap of PbS QDs, suggesting the response behavior of device combined the merits of two materials. The *I–T* characteristics under the illumination of 350 nm have illustrated above. Herein, it is worth to note that the device exhibits stable and fast response under 500 and 900 nm irradiation. During the six cycles of the on–off test at the three wavelengths, which lasted over 350 s, the photocurrent increased to a stable value, and then dramatically decreased to its initial value as the light was turned off, confirming the robustness of the metal oxide NWs and QDs. The *t*_r_ and *t*_d_ for the two wavelengths of light are calculated to be less than 0.5 s both. As the limitation of measuring accuracy of our instruments, actual rise and decay time might be faster. The vertical rise and drop of current with light on and off indicate that the device is ultrasensitive to 500 and 900 nm light. The detectivity for 500 and 900 nm was also calculated, which can reach 4.9 × 10^7^ and 7.2 × 10^7^ Jones, respectively. These results demonstrated that the response range for ZnO NW is broadened to NIR after decorating PbS QDs even though these indexes are not as well as reported p–n junction photodiodes based on PbS QD film and ZnO layers.^53^ As ZnO NWA hardly has response to 500 and 900 nm as shown in Figure S7 (Supporting Information), the photocurrent is close to the dark current, and the current was not changed when switching on and off the light. This conclusion could be verified by the photoluminescence of ZnO as shown in Figure S8 (Supporting Information), only a luminescence band centered at 380 nm corresponding to near‐band‐edge (3.37 eV) emission. The photoresponses for PbS QDs at 500 and 900 nm light were also described in Figure S9 (Supporting Information). The rise and decay time for PbS QDs were less than 0.5 s, however, the photocurrent is even lower than ZnO/PbS QDs heterostructure under the same wavelength and bias. We can infer that a better photoresponse for heterosturcture at 500 and 900 nm is due to the photoexcitation of PbS QDs in the vis and NIR regime, the photogenerated electrons will transfer to the ZnO NW, leading to a separation of electrons and holes.^21^ In short, the ZnO/PbS heterostructure has a much faster response speed than that of ZnO in the UV range, and possesses a wider spectral response reaching to the NIR range.

### 2.3 Flexibilities of ZnO/PbS NWF in UV–Vis–NIR Range

**Figure 5:**
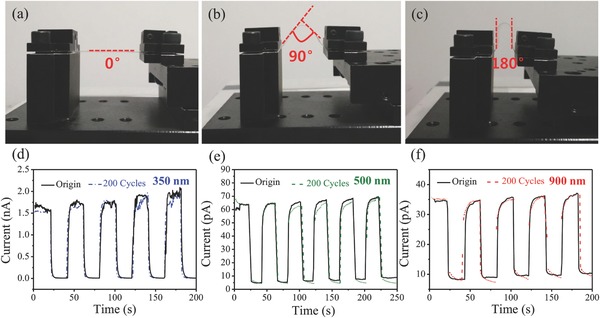
a–c) Optical images of the flexible samples on mica substrate at various bending degree. d–f) *I*–*T* curves between the original sample and the sample subjected to 200 bending cycles under the illumination of 350, 500, 900 nm light, respectively, all the bending degree are 180°.

ZnO NW‐based photodetectors have great potential for flexible photodetectors^54^, ^55^ which are important functional components for light‐weight, flexible optoelectronics,^56^, ^57^ however, high‐performance flexible NW photodetectors with UV to NIR response are still need to be developed.^58^ Benefiting from the flexibility of mica sheets and heterogeneous ZnO/PbS QDs thin film, flexible photodetectors based on ZnO/PbS QDs heterostructure were fabricated on mica by traditional device fabrication techniques as shown in the Experimental Section. Photograph of set‐up was shown in Figure S10 (Supporting Information). Combined with the excellent flexibility of mica substrate, the photoresponse of the device under bending was well maintained. Optical images of the flexible samples bending at 0°, 90°, 180° demonstrate a bendable performance in **Figure** **5**a–c. The photoresponse of the device is obtained under the illumination of 350, 500, and 900 nm light. Surprisingly, the photoresponse of the NWF was almost unchanged for the UV–vis to NIR irradiation when bending to 180° or even under repeated 200 cycles, as demonstrated in Figure 5d–f, respectively, indicating that bending stress can hardly influence the photoelectric performance of the device. The photo–dark current ratio and response (rise/decay) speed of the NWF were well maintained after 180° bending. These results clearly demonstrate the excellent photoelectrical stability of this heterostructure under extreme bending conditions. Moreover, Figure S11 (Supporting Information) also exhibits the *I*–*V* properties of the devices exposed with 350, 500, and 900 nm light before and after bending for 200 cycles. We can clearly observe that after bending for 200 cycles, there was negligible change in the photocurrent of the flexible device, and the ohmic contact kept well, revealing the outstanding deformation tolerance of the flexible ZnO/PbS QDs device.

## 3 Application to Other NWs and QDs

**Figure 6:**
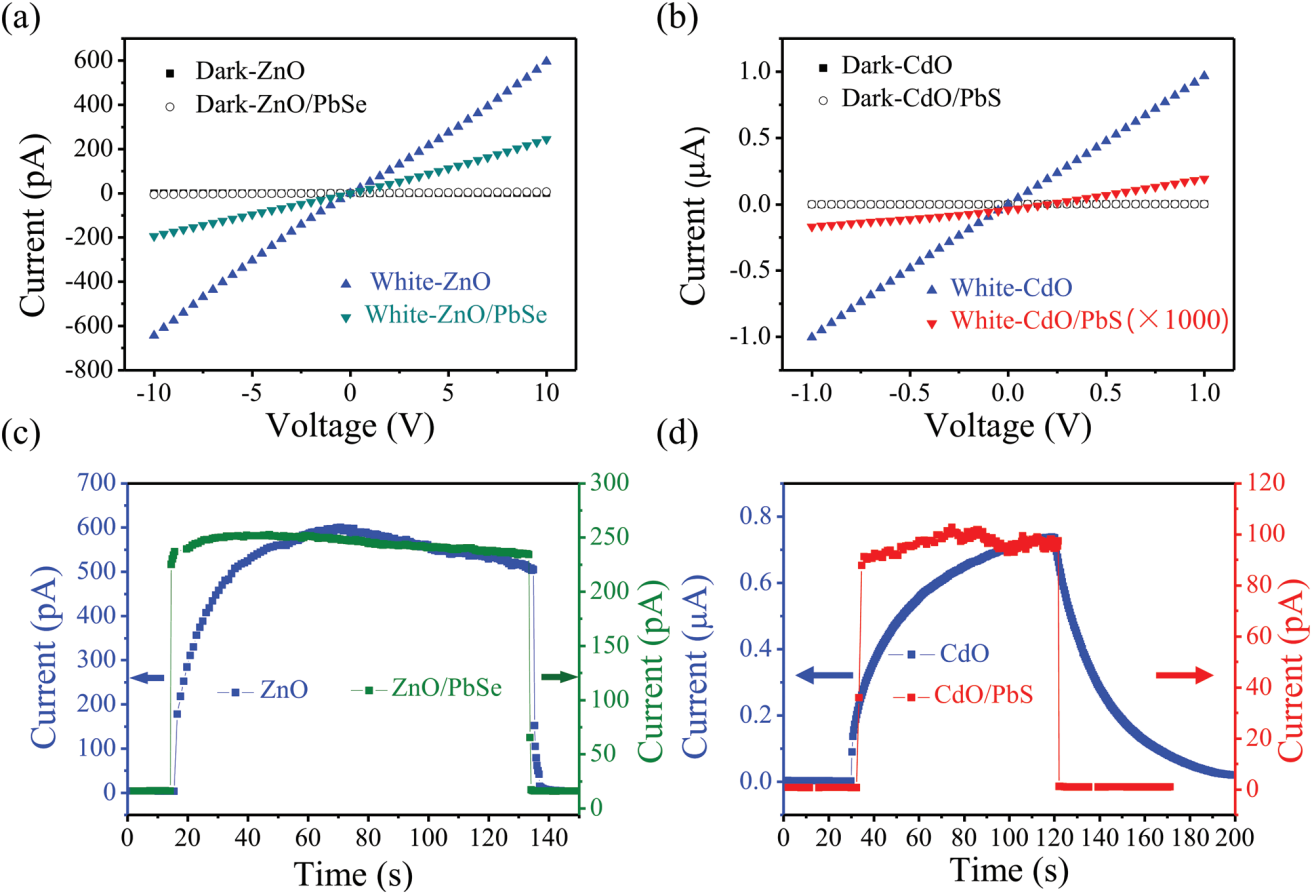
a,b) *I*–*V* curve for ZnO/PbSe, CdO/PbS heterostructures in the dark and under the illumination of white light. c,d) *I*–*T* curve for ZnO/PbSe, CdO/PbS heterostructures in the dark and under the illumination of white light.

As‐fabricated solution of QDs are typically capped with long ligands (oleic acid or oleylamine) which are necessary to maintain colloidal stable in the organic solvents,^59^ therefore, it is not easy to attach the QDs onto nanowires. Basically, ZnO NWs should be decorated with positive charged functional groups such as 3‐aminopropyltrimethoxysilane^60^ or treated the NWs by oxygen plasma^61^ to facilitate the attachment of QDs on NWs, then an EDT or TBAI solution was used to exchange the long ligand. Herein, we demonstrate that this EDT exchanged method for forming the heterojunction could be expanded to other OA encapsulated QDs such as PbSe, and other NWA such as CdO by this modified electrospinning strategy. Optical photographs of ZnO/PbSe QDs, CdO/PbS QDs devices are shown in Figure S3b,c (Supporting Information), respectively. To verify the enhanced response of compound, white light is used to excite the heterojunction, variation of *I–V* and *I–T* curves after composition is shown in **Figure** **6**. Totally, the contacts between NWA and electrodes are ohmic, indicating the high quality of photodetectors, moreover, the ohmic contacts were maintained after compositing with QDs. Reason for decreasing of the photocurrent could be explained the same as ZnO/PbS QDs heterojunction. Surprisingly, three kinds of heterojunctions demonstrate nearly the same improvement of rise and decay speed, both *t*_r_ and *t*_d_ are less than 0.5 s. Comparison of rise speed and decay speed for ZnO/PbSe QDs, CdO/PbS QDs heterostructure photodetectors is listed in **Table** **1**, respectively. The response speed for pure NWA is slow, for example, CdO, the rise and decay time are as long as 54 and 52 s, respectively, which could be attributed to the polycrystalline structure of the NWs and porous morphology induced by burning off of polymer. These NWs made up of agglomerate nanoparticles may have large specific surface area, the photoresponse is thus mainly dominated by various surface processes, i.e., O_2_ adsorption and desorption, which is usually very slow.^48^ This response time was significantly decreased to less than 0.5 s after decorating PbS QDs, as shown in Figure 6d. Based on the analysis above, this ligand exchange mechanism may provide a strategy for compositing of 0D and 1D nanomaterials, which have great potential application in the future nanofabrication and nanoprocessing.^62^

**Table 1:** Comparison of rise speed and decay speed for ZnO/PbSe, CdO/PbS heterojunction photodetectors, respectively

Photodetector	Light	Rise time (pure NW)	Rise time (heterostructure)	Decay time (pure NW)	Decay time (heterosturcture)
ZnO/PbSe	White	40 s	<0.5 s	2 s	<0.5 s
CdO/PbS	White	54 s	<0.5 s	52 s	<0.5 s

## 4 Conclusions

We have demonstrated an enhanced UV–vis–NIR photodetector based on electrospun ZnO NWA/PbS QDs film heterostructures that are constructed via a simple ligand exchange operation. Remarkably, this heterostructure based photodetectors showed an enhanced response speed in UV range and high stability in UV–vis–NIR range. Moreover, these photodetectors fabricated on mica demonstrated good flexibility which showed a stable photoresponse even after 200 cycles 180° bending, indicating an excellent photoelectrical and mechanical stability. This electrospinning and ligand exchange process is simple and effective, which could also be applied to other 1D and 0D materials heterostructures like ZnO/PbSe QDs and CdO/PbS QDs. We expected this strategy would be beneficial to the development of future UV–vis–NIR and flexible optoelectronic circuitry.

## 5 Experimental Section

*Fabrication of ZnO NWA*: ZnO NWA were synthesized based on a modified electrospinning set*‐*up (SS–2535H, Ucalery), as introduced before.^63^ In brief, 1.2 g of polyvinylpyrrolidone (PVP) powder (*M*_n_ = 13 00 000) was dissolved in 10 mL of N,N–dimethylformamide (DMF) solution. Then, 1.5 g of Zn(CH_3_COO)_2_⋅2H_2_O were added into the PVP/DMF solution to form a uniform liquid as the source. A high positive 10 kV bias was applied to the needle tip of the syringe, whereas the other terminal which connected to two rectangular aluminum foil contacts with a channel width of 10 mm was applied with negative 3 kV bias. The distance between the needle tip and the collector was set as 15 cm. As a result, uniaxially aligned NWs of one layer thickness were obtained in the gap. Afterward, the NWs were calcined in air at a rate of 5 °C min^−1^ and kept for 3 h at 500 °C.

*Device Fabrication*: For the NWA device, the electrodes (Ti/Au (10/50 nm)) were deposited onto the quartz substrate via electron beam evaporation (Nexdep, Angstrom Engineering) using an Al foil as a shadow mask.

*Spin PbS QDs and Ligand Exchange Using EDT*: The fabricated PbS QDs^64^ solution was spinning onto the ZnO NWA device at the speed of 4000 rpm. This process was repeated 5–10 times until the QDs were linked to a film. Then 1 mmol L^−1^ EDT/acetonitrile solution was dropped on the device and maintained for two minutes. At last, the exchanged device was rinsed by the dimethyl sulfoxide.

*Characterization and Measurements*: The morphology of as‐prepared ZnO/PbS heterogeneous arrays was characterized by an optical microscope (BX51, OLMPUS), and TEM (Tecnai G2 F30, FEI and JEM 2100, JEOL) equipped with EDS was used to characterize the microstructure. Photoluminescence spectra of ZnO nanowires were collected with a He‐Cd laser (LabRAM HR800, Horiba JobinYvon). *I*–*V* and *I*–*T* characteristics were measured by a low‐temperature cryogenic probe station (CRX–6.5K, Lake Shore) and a semiconductor parameter analyzer (4200‐SCS, Keithley). A laser‐driven white‐light source (EQ‐1500, Energetiq) was used for the measurements of wide‐range spectral runs between 300 and 1000 nm, respectively. The light intensity was calibrated using a UV‐enhanced Si photodiode.

## Supporting information

As a service to our authors and readers, this journal provides supporting information supplied by the authors. Such materials are peer reviewed and may be re‐organized for online delivery, but are not copy‐edited or typeset. Technical support issues arising from supporting information (other than missing files) should be addressed to the authors.

Supplementary
