# Supplementary material for: An Enhanced UV–Vis–NIR an d Flexible Photodetector Based on Electrospun ZnO Nanowire Array/PbS Quantum Dots Film Heterostructure
Source: Adv Sci (Weinh). 2016 Dec 30;4(3):1600316. doi: 10.1002/advs.201600316 (PMC5357981; doi:10.1002/advs.201600316)
Supplement: Supplementary file 1 — Supplementary [file ADVS-4-na-s001.pdf]

Copyright WILEY-VCH Verlag GmbH & Co. KGaA, 69469 Weinheim, Germany, 2016.

ADVANCED SCIENCE
Open Access

Supporting Information

for *Adv. Sci.,* DOI: 10.1002/advs.201600316

An Enhanced UV–Vis–NIR an d Flexible Photodetector Based on Electrospun ZnO Nanowire Array/PbS Quantum Dots Film Heterostructure

*Zhi Zheng, Lin Gan, Jianbing Zhang, Fuwei Zhuge, and Tianyou Zhai**

Copyright WILEY–VCH Verlag GmbH & Co. KGaA, 69469 Weinheim, Germany, 2016.

Supporting Information

An Enhanced UV-Vis-NIR and Flexible Photodetector Based on ZnO Nanowire Array/ PbS Quantum Dots Heterojunction

Zhi Zheng, Lin Gan, Jianbing Zhang, Fuwei Zhuge, and Tianyou Zhai*

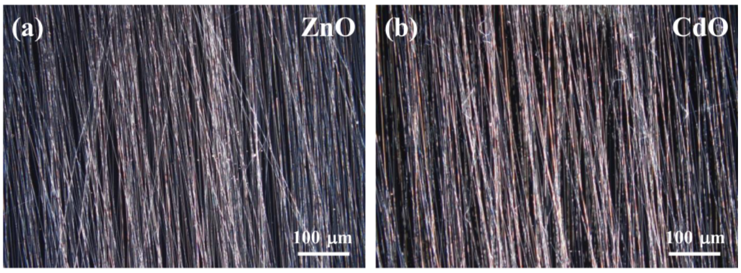

Figure S1. a) Optical photograph for ZnO NWA. b) Optical photograph for CdO NWA.

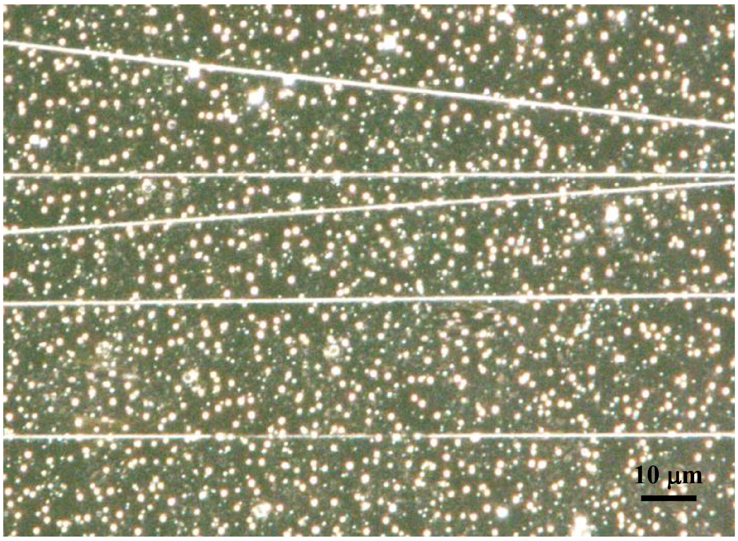

Figure S2. Optical photograph for ZnO NWA/PbS QDs hybrid thin film.

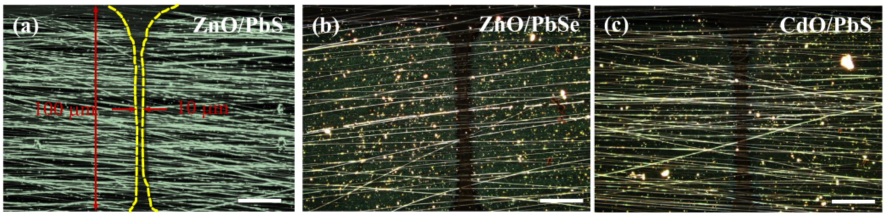

Figure S3. a) Optical photograph of ZnO NWA and PbS QDs thin film device, the yellow dotted line refers to the boundary of electrodes. b) Optical photograph of ZnO NWA and PbSe QDs thin

film device. c) Optical photograph of CdO NWA and PbS QDs thin film device. Scale bar is 100 µm.

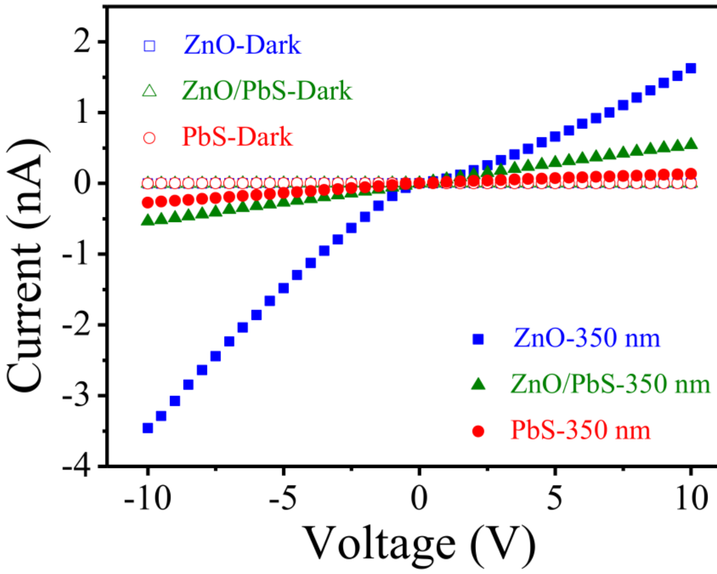

Figure S4. I–V curves of the ZnO, ZnO/PbS, PbS device illuminated with light of 350 nm wavelengths and in the dark.

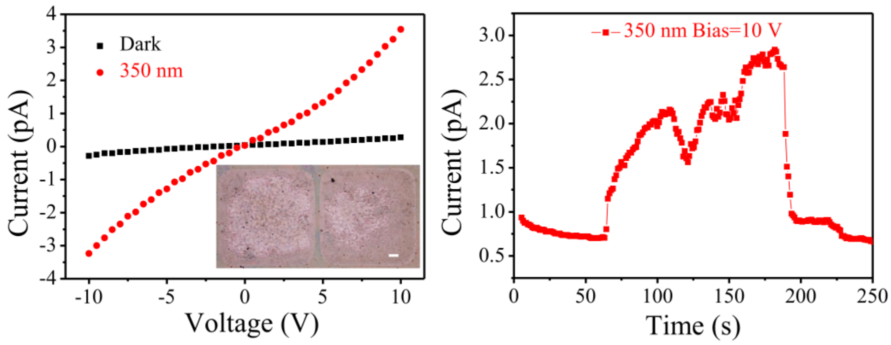

Figure S5. a) I–V curves of the PbS QDs thin film devices illuminated with light of 350 nm, inset is the optical image of this device; scale bar is 50µm. b) I–T curves devices during on-off switching tests under 350 nm illumination at the bias of 10 V.

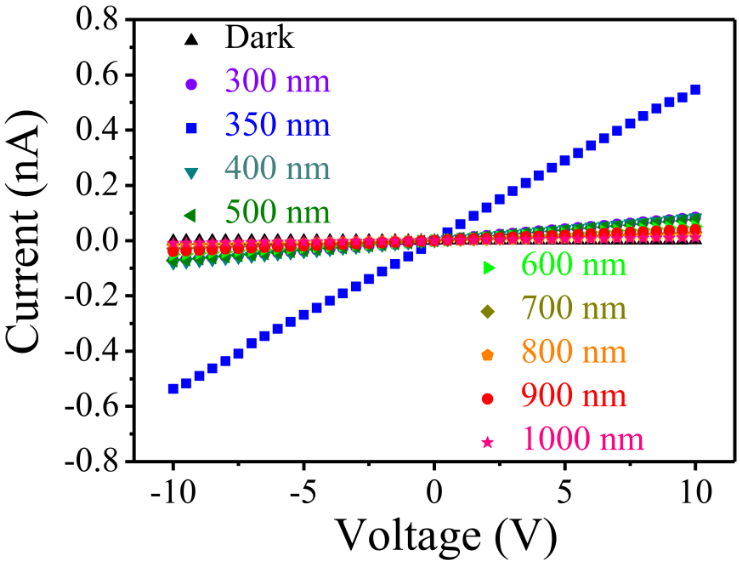

Figure S6. I–V curves of the device illuminated with light of 300 nm, 350 nm, 400 nm, 500 nm, 600 nm, 700 nm, 800 nm, 900 nm, 1000 nm wavelengths and in the dark.

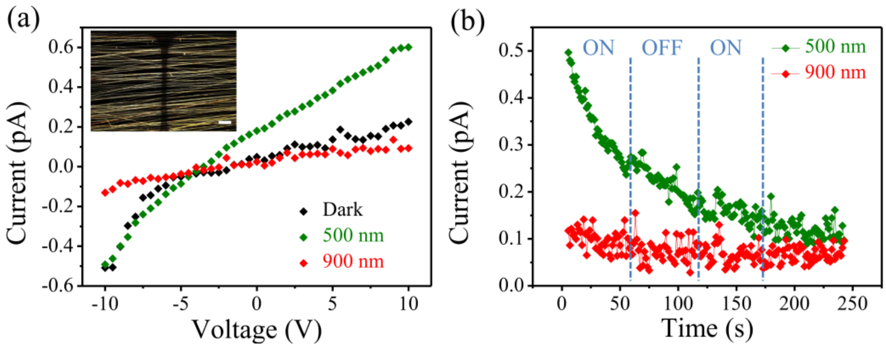

Figure S7. a) I–V curves of the ZnO NWA devices illuminated with light of 500 nm and 900 nm, inset is the photograph for this device; scale bar is 50 μm. b) *I–T* curves devices during on-off switching tests under 500 nm, and 900 nm illumination at the bias of 10 V.

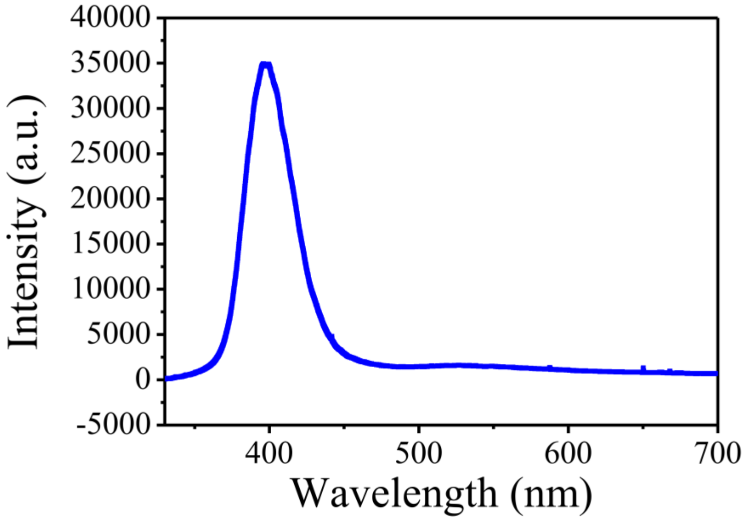

Figure S8. Photoluminescence (PL) spectra of the ZnO nanowire samples, collected with a He-Cd laser (325 nm line).

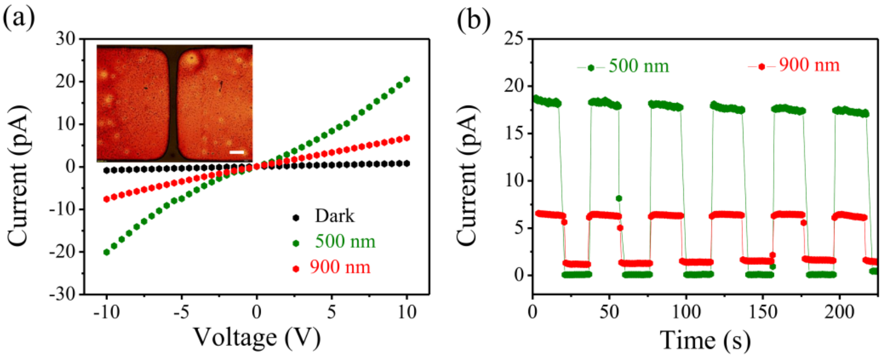

Figure S9. a) I–V curves of the PbS QDs thin film devices illuminated with light of 500 nm and 900 nm, inset is the photograph of the device, scale bar is 50 µm. b) *I–T* curves devices during on-off switching tests under 500 nm, and 900 nm illumination at the bias of 10 V.

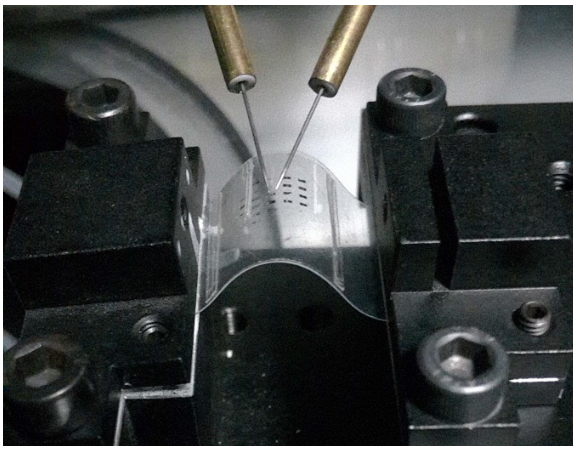

Figure S10. Optical micrograph of on-line bending the samples on mica substrate.

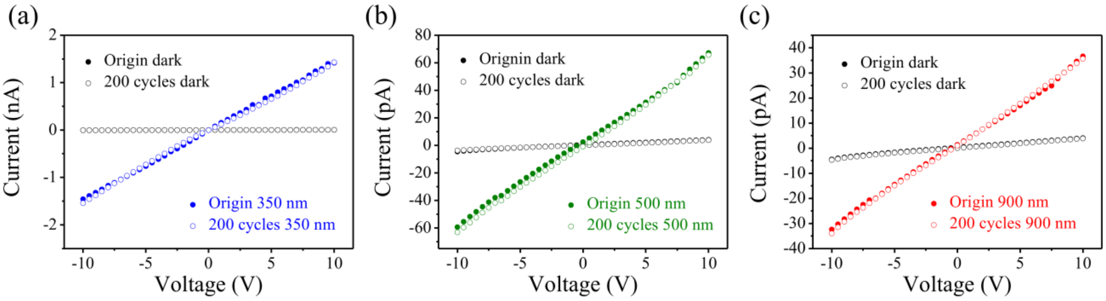

Figure S11. a, b, c) *I–V* curves between the original sample and the sample subjected to 200 bending cycles under the illumination of 350 nm, 500 nm, and 900 nm light, respectively.
